# IL-33/ST2 Pathway and Classical Cytokines in End-Stage Heart Failure Patients Submitted to Left Ventricular Assist Device Support: A Paradoxic Role for Inflammatory Mediators?

**DOI:** 10.1155/2013/498703

**Published:** 2013-12-10

**Authors:** C. Caselli, A. D'Amico, R. Ragusa, R. Caruso, T. Prescimone, M. Cabiati, S. Nonini, P. Marraccini, S. Del Ry, M. G. Trivella, O. Parodi, D. Giannessi

**Affiliations:** ^1^Laboratory of Cardiovascular Biochemistry, Institute of Clinical Physiology, Consiglio Nazionale delle Ricerche (CNR), Area della Ricerca, Via Moruzzi 1, 56100 Pisa, Italy; ^2^Institute of Life Sciences, Scuola Superiore Sant'Anna, 56100 Pisa, Italy; ^3^Cardiovascular Department, Institute of Clinical Physiology, Consiglio Nazionale delle Ricerche (CNR), Niguarda Cà Granda Hospital, 20162 Milan, Italy; ^4^Cardiotoracovascular Department, Niguarda Cà Granda Hospital, 20162 Milan, Italy

## Abstract

*Background*. Inflammation is a critical process contributing to heart failure (HF). We hypothesized that IL-33/ST2 pathway, a new mechanism regulated during cardiac stress, may be involved in the functional worsening of end-stage HF patients, candidates for left ventricular assist device (LVAD) implantation, and potentially responsible for their outcome. *Methods*. IL-33, ST2, and conventional cytokines (IL-6, IL-8, and TNF-**α**) were determined in cardiac biopsies and plasma of 22 patients submitted to LVAD implantation (pre-LVAD) and compared with (1) control stable chronic HF patients on medical therapy at the moment of heart transplantation without prior circulatory support (HT); (2) patients supported by LVAD at the moment of LVAD weaning (post-LVAD). *Results*. Cardiac expression of ST2/IL-33 and cytokines was lower in the pre-LVAD than in the HT group. LVAD determined an increase of inflammatory mediators comparable to levels of the HT group. Only ST2 correlated with outcome indices after LVAD implantation. *Conclusions*. IL-33/ST2 and traditional cytokines were involved in decline of cardiac function of ESHF patients as well as in hemodynamic recovery induced by LVAD. IL-33/ST2 pathway was also associated to severity of clinical course. Thus, a better understanding of inflammation is the key to achieving more favorable outcome by new specific therapies.

## 1. Introduction 

Inflammation has emerged as a critical biological process contributing to nearly all aspects of cardiovascular disease including heart failure (HF) [1]. Several studies have identified the importance of proinflammatory mediators (such as TNF-*α*, IL-6, and IL-8) in the development and progression of HF [2–4]. These factors can induce pathological myocardial remodeling by promoting the recruitment of inflammatory cells or by producing maladaptive effects in the heart on left ventricle (LV) function, such as LV remodeling and endothelial function, thus facilitating hypertrophic growth and fibrosis [5]. The recognition of inflammation as a common, important, and treatable condition in chronic HF has contributed to intensified research for an effective anti-inflammatory therapy. However, the approaches tested so far have been largely disappointing, due to either neutral findings or a worsening of HF. These discouraging results have raised important considerations, including the relevance of looking for novel targets evolving from basic research [6].

Recently, a number of studies have used gene expression, array screening, cloning, and other techniques to identify new cardiokines and cardiokine networks that are regulated during cardiac stress [4]. With mouse genetic approaches, many of these newly identified factors have been shown to have functional roles in pathological cardiac remodeling. Interleukin (IL)-33 (also known as IL-1F11), an IL-1 family member [7], binds to a ST2L (also known as T1, IL-1RL1, and DER4), which is a member of the Toll-like receptor (TLR)/IL1R superfamily. The ST2 gene can encode at least two other isoforms in addition to ST2L by alternative splicing, including a secreted soluble ST2 (sST2) form which can serve as a decoy receptor for IL-33 and an ST2V variant form [8]. Studies in animal models suggest that IL-33/ST2 is implicated in cardiovascular disease and signaling as an important protective pathway in various heart diseases including HF [9–11].

Despite significant progress in the treatment of HF, patients with advanced HF continue to present significant morbidity and mortality. As HF reaches end stage, the only viable options currently available are transplantation, mechanical circulatory support such as left ventricular assist device (LVAD), or terminal care [12–14]. LVAD provides profound volume and pressure unloading of the left ventricle and restores systemic blood pressure and flow to near normal levels, leading, after long-term support, to a normalization of neurohormonal and cytokine levels that contributes to myocardial recovery [15, 16]. LVAD provides a unique model with which to gain insight into both adverse and reverse remodeling [1].

Our hypothesis is that the myocardial IL-33/ST2 pathway may be involved in the hemodynamic disarrangement in end-stage HF (ESHF) patients, candidates for LVAD implantation, and may be potentially responsible for their outcome. To investigate this hypothesis, the inflammatory phenotypic profile of patients undergoing LVAD implantation will be evaluated, taking into account both the IL-33/ST2 pathway and conventional inflammatory biomarkers such as IL-6, IL-8, and TNF-*α*. A better characterization of inflammatory cytokines, including IL-33/ST2 pathway, will lead to a better mechanistic understanding of HF and could identify new diagnostic and therapeutic targets for HF. Particularly, specific aims were:

1. to evaluate the role of inflammation in HF patients undergoing LVAD implantation (pre-LVAD group), using a group of stable HF patients undergoing heart transplantation (HT group) as control,
2. to evaluate the effect of LVAD support on inflammation comparing the pre-LVAD group with patients at the time of LVAD weaning (post-LVAD group),
3. to evaluate the role of inflammation in early outcomes in a pre-LVAD group of patients.

## 2. Materials and Methods 

### 2.1. Patients and Study Design

A total of twenty-two ESHF patients who underwent LVAD implantation as bridge to heart transplantation were enrolled in the study (pre-LVAD group). All patients were supported by axial continuous-flow devices (16 were HeartMateII LVADs (Thoratec, Pleasanton, CA), 4 were Incor LVADs (Berlin Heart AG), 1 was De Bakey LVADs (MicroMed Technology, Inc., Houston, TX), and 1 was HeartWare LVAD (HeartWare International Inc., Framingham, MA)). Cardiac biopsies were obtained at the moment of LVAD implantation and plasma samples were collected at preimplant and subsequently up to 1 month since LVAD implantation. Multiorgan function was monitored preoperatively and until 2 weeks after LVAD implantation, calculating the total Sequential Organ Failure Assessment (tSOFA) score. The SOFA system is a daily score from 0 to 4 assigned in proportion to the severity of functional deterioration for each six individual organ system (cardiovascular, respiratory, hepatic, renal, neurological, and hemocoagulative) [17]. The clinical course of these patients was assessed considering the following end points: tSOFA score at 1 week, length of intensive care unit (ICU) stay, hospitalization, and 3-month survival. The combination of postoperative tSOFA score ≥11 and/or ICU death was taken into account as main composite adverse outcome during ICU stay.

In order to evaluate the inflammatory condition in ESHF patients and the effect of LVAD support, inflammatory mediators determined in pre-LVAD group of patients were compared with further two different groups of patients:

*(1) HT Group (Control)*. Cardiac samples were collected at the moment of heart transplantation from seven stable chronic HF patients on medical therapy, without prior circulatory support. 

*(2) Post-LVAD Group*. Cardiac biopsies were obtained from six patients supported by LVAD implanted as a bridge to heart transplantation at the moment of LVAD weaning. Five LVAD patients were supported by axial continuous-flow devices (3 were De Bakey LVAD (MicroMed Technology Inc, Houston, MT, USA), and 2 were HeartMateII LVAD (Thoratec, Pleasanton, CA, USA)), whereas 1 patient was supported by a pulsatile-flow device (NewCorTec, Rome, Italy).

The study conformed the principles outlined in the Declaration of Helsinki and the study protocol was approved by local ethics committee. All subjects gave written informed consent to participate to the study.

### 2.2. Biological Samples

Cardiac biopsies from the pre-LVAD group were collected at the time of LVAD implantation from the portion of LV apex excised during standard surgical procedure (needed for inflow cannula positioning). Five myocardial samples from post-LVAD and HT groups were collected at the time of transplantation from the similar areas of left (LV) and right ventricle (RV), as previously reported [18]. Sample collection resulted in *n* = 22 myocardial samples in the pre-LVAD group, *n* = 30 in the post-LVAD group, and *n* = 35 in the HT group. Immediately after collection, myocardial samples were frozen in liquid nitrogen and stored at –80°C until sample preparation.

Blood samples of pre-LVAD group were obtained before and 1 month after LVAD implantation. Plasma specimens were collected in tubes with EDTA and then separated by centrifugation for 15 min at 1000 ×g. The plasma was stored at −20°C and the specific immunometric assay was performed within 2 months after collection.

### 2.3. RNA Preparation

Total RNA was extracted from cardiac samples by acid guanidinium thiocyanate-phenol-chloroform method using Rneasy Midi kit (Qiagen S.p.a, Milano, Italy). RNA concentration was evaluated spectrophotometrically (BioPhotometer; Eppendorf Italia, Milan, Italy) and RNA purity by electrophoresis of samples on Gel Star Stain (Lonza, Rockland Inc., ME, USA) agarose gels. RNA samples were stored at −80°C for use in gene expression studies.

Following DNAse treatment (RNase-Free DNase Set, Qiagen S.p.a), first-strand cDNA was synthesized by iScript cDNA Synthesis kit (Bio-Rad) starting from about 1 *μ*g total RNA as template. Reverse transcriptase reaction consisted of an incubation step at 25°C for 5 min, followed by three different cycles at 42°C for 30 min and 45–48°C for 10 min. The reverse transcriptase enzyme was inactivated by heating to 85°C for 5 min. The cDNA samples obtained were placed on ice and stored at 4°C.

### 2.4. Real-Time PCR

Novel inflammatory mediators, including IL-33 and ST2, and traditional molecules, such as IL-6, IL-8, and TNF-*α*, were studied. Primer Express Version 2.0 (Applied Biosystems) was used for designing primers. Real-time PCR reactions were performed in duplicate by the Bio-Rad C1000 thermal cycler (CFX-96 real-time PCR detection systems; Bio-Rad). In order to monitor cDNA amplification, Eva-Green (SsoFAST EvaGreen Supermix; Biorad), was used as fluorophore. Reactions (20 *μ*L) contained 2 *μ*L template cDNA, 0.2 *μ*M forward and reverse primers, 1x SsoFAST EvaGreen SuperMix (Bio-Rad), and sterile H_2_O. The cycling condition included an initial denaturation-activation step at 98°C for 30 s, followed by 40 cycles at 95°C for 5 s and 60°C for 30 s. Multiple interrun calibrators were always used to allow comparison of Ct values obtained in different runs.

In order to normalize the inflammatory gene expression, genes previously selected [18] and confirmed in these set of samples were used as reference. The reaction conditions of all primer pairs were assessed determining the optimal annealing temperature, conducting a gradient PCR, and to verify efficiency, generating a standard curve obtained by scalar dilution of a cDNA pool. To increase the reliability and integrity of study results and to promote the effort for experimental consistency and transparency between research laboratories, we adhered to the minimum information for publication of quantitative real-time PCR experiments (MIQE) guidelines [19]. Accordingly, the characteristics of each selected primers were reported in Table 1.

### 2.5. Plasma Determination

Circulating levels of sST2 and IL-33 were assessed in plasma samples by specific enzyme-linked immunosorbent assays (R&D Systems, Minneapolis, MN-USA for IL-6 and IL-8). Inter-assay and intraassay CVs were <10%.

### 2.6. Statistical Analysis

Data are expressed as median and interquartile range (I–III). Expression differences between patient groups were assessed by Mann-Whitney *U* test. Differences of plasma levels of cytokines between before and after 1 month LVAD support were assessed by Wilcoxon signed-rank tests for which Bonferroni correction resulted in a significance level set at  *P* < 0.05. For correlation analysis, skewed variables were logarithmically transformed to improve normality before analysis, and Pearson's correlation was used to analyze the relationship between variables. A 2-tailed  *P* value < 0.05 was considered statistically significant. Statistical analysis was carried out using the SPSS v19 (1989–2010, SPSS Inc., Chicago, IL, USA).

## 3. Results

### 3.1. Clinical Characteristics of Patients

The clinical characteristics of the patients are described in Table 2. Clinical features were compared according to the previously described experimental groups (pre-LVAD, HT, and post-LVAD group).

#### 3.1.1. LVAD Candidates and HT Group

Median age of LVAD candidates (pre-LVAD group) was comparable to that of patients who underwent elective HT on medical therapy, without prior circulatory support (HT group). The idiopathic dilatative cardiomyopathy (IDC) was prevalent in both groups. Echocardiographic parameters as well as medical therapies did not differ between pre-LVAD and HT patients; antiplatelet and anticoagulant agents, which were mandatory in pre-LVAD patients, were prevalent in pre-LVAD group. Total bilirubin and creatinine values did not show differences between pre-LVAD group and HT group.

#### 3.1.2. LVAD Candidates and LVAD Patients Prior Heart Transplantation

Among post-LVAD group, the median support time prior to heart transplantation was 367 (152–483) days. Median age of patients of post-LVAD group was lower than that of patients LVAD candidate. At heart transplantation, in patients of post-LVAD group, the levels of cardiac index, right atrial pressure, pulmonary capillary wedge pressure, and NT-proBNP were lower than those of pre-LVAD group and comparable to those of patients of HT group.

#### 3.1.3. Postoperative LVAD Outcome

After LVAD implantation, all pre-LVAD group of patients experienced postoperative hemodynamic improvement with respect to that at preimplant (data not shown). At 3 postoperative months, 4 out of 22 (18%) pre-LVAD group of patients had died, in particular during ICU stay (second and third postoperative week), with multiorgan failure syndrome (MOFS) as main cause of death. Among survivors, the ICU length of stay was of 14 (9–23) days, while hospitalization was of 45 (30–67) days.

In all patients, the tSOFA score at 1 postoperative week was higher than that at preimplant (9 (4–10) and 4 (2–5), resp.,  *P* = 0.001). However, eight patients experienced severe multiorgan failure evidenced by postoperative tSOFA score ≥11. Overall, nine of 22 patients (41%) experienced postoperative tSOFA score ≥11 and/or ICU death, together considered as composite critical outcome.

### 3.2. Inflammatory Condition of ESHF Patients at Baseline

The evaluation of inflammatory condition of cardiac muscle was checked in ESHF patients at the moment of LVAD implantation (pre-LVAD group) compared with a group of stable HF patients submitted to heart transplantation as control (HT group).

**Figure 1:**
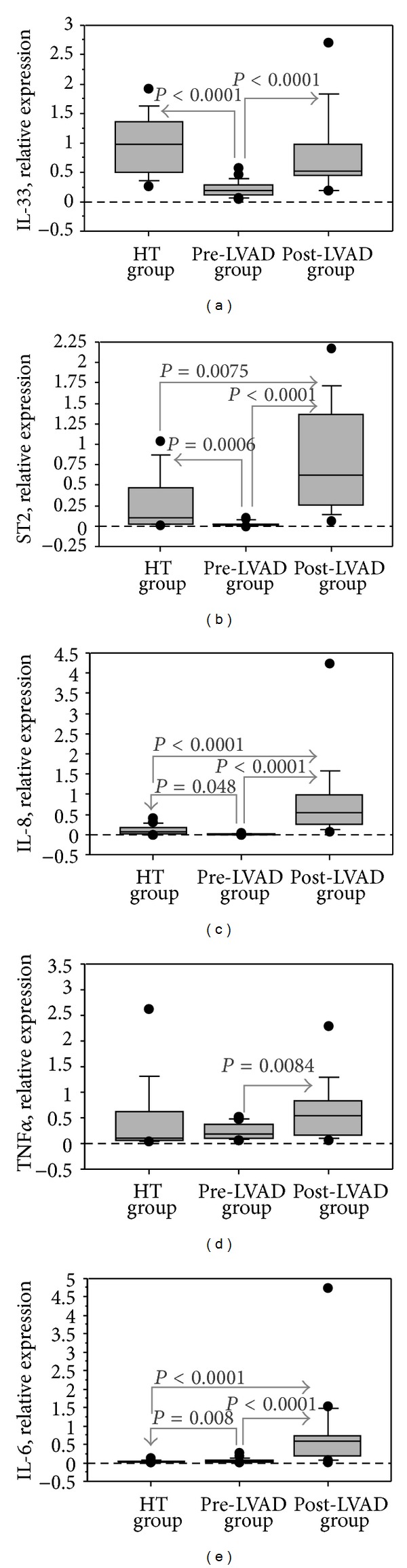
Regulation of mediators of inflammation in cardiac tissue from ESHF patient of pre-LVAD group, HT control group, and post-LVAD group. Relative quantification of IL-33 (a), ST2 (b), IL-8 (c), TNF-*α* (d), and IL-6 (e) is shown.

Cardiac expression of IL-33 and ST2, the novel inflammatory pathway, was significantly lower in pre-LVAD group than in control group (HT group) (Figures 1(a) and 1(b)). Similarly, the levels of classical mediators (IL-8 and TNF-*α*) were significantly lower in pre-LVAD group than in HT group (Figures 1(c) and 1(d)), except for IL-6 that showed significantly higher levels (Figure 1(e)).

**Figure 2:**
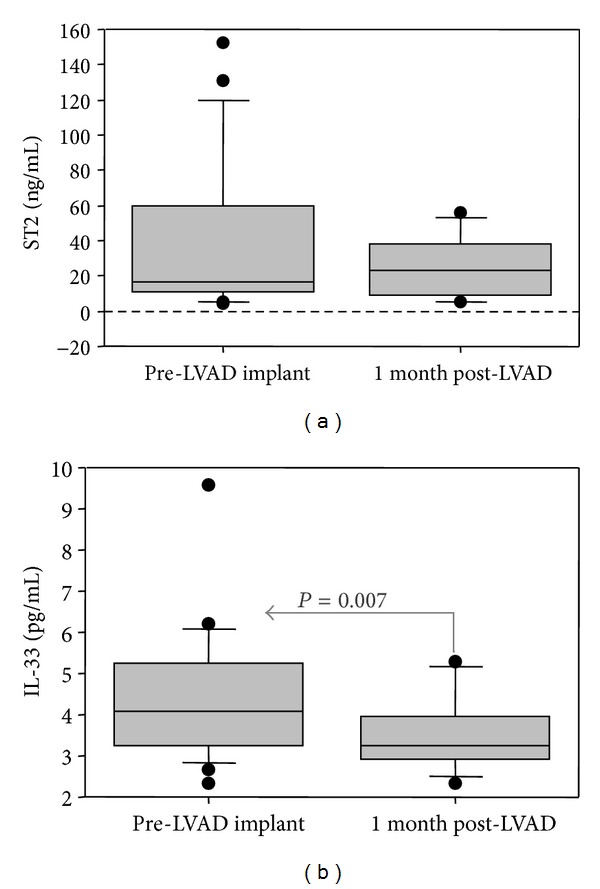
Effect of 1 month LVAD support on sST2 (a) and IL-33 (b) circulating levels.

Circulating levels of inflammatory mediators were reported in Figure 2. Cardiac ST2 expression was positively associated with circulating sST2 levels (*r* = 0.771, 95% CI 0.354 to 0.932,  *P* = 0.003). Conversely, cardiac IL-33 expression was negatively related to circulating IL-33 levels (*r* = −0.613, 95% CI −0.878 to −0.061,  *P* = 0.034).

**Figure 3:**
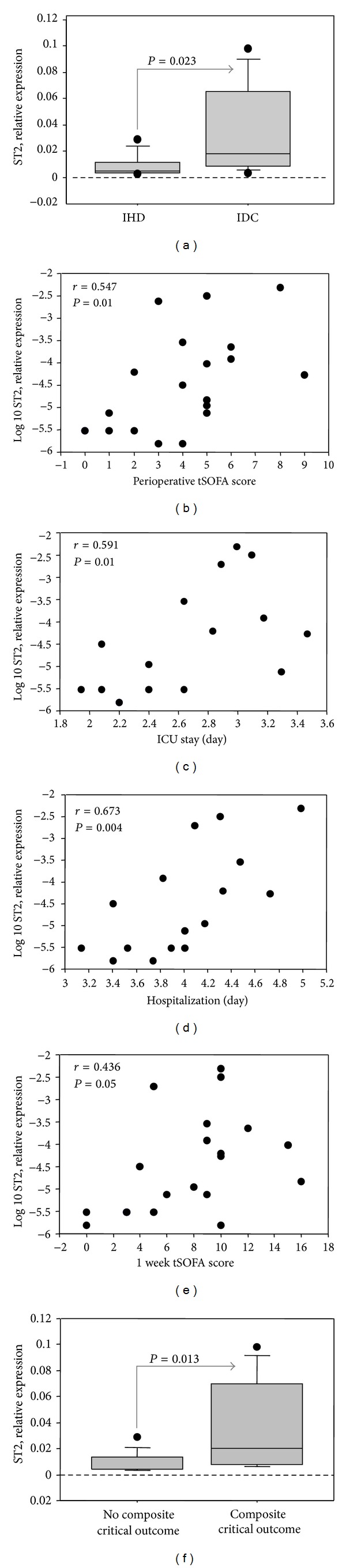
Relation between myocardial ST2 expression and both diagnosis ((a) and (b)) and outcome ((c) and (d)) of pre-LVAD group of samples. (a) Different ST2 expression according to HF etiology; (b) correlation with perioperative tSOFA score; (c) correlation with duration of ICU stay; (d) correlation with length of hospitalization; (e) correlation with 1 week tSOFA score; (f) ST2 expression according to composite outcome.

Among clinical features of pre-LVAD group, etiology of HF was identified by cardiac ST2 levels, being significantly higher in IDC than IHD patients (Figure 3(a)). Again, only myocardial ST2 showed a positive correlation with perioperative tSOFA score (Figure 3(b)) and plasma levels of its soluble isoform sST2 tended to be correlated with tSOFA score (*r* = 0.449, 95% CI −0.107 to 0.791,  *P* = 0.107).

### 3.3. Changes of Inflammatory Profile after LVAD Implant

The effect of LVAD support on inflammatory status was evaluated at tissue level by comparison of pre-LVAD group with a group of patients at the time of LVAD weaning (post-LVAD group). Both novel and traditional inflammatory mediators were significantly higher in post-LVAD group compared with pre-LVAD group (Figure 1). Moreover, the cardiac levels of IL-33 and TNF-*α* in post-LVAD group were comparable with their respective levels in HT group (Figures 1(a) and 1(d)), while the cardiac levels of ST2, IL-6, and IL-8 were significantly higher in post-LVAD group than HT group (Figures 1(b), 1(c), and 1(e)). Only cardiac ST2 correlated with cardiac IL-33 (*r* = 0.520, 95% CI 0.512 to 1.880,  *P* = 0.001), IL-6 (*r* = 0.688, 95% CI 0.637 to 1.376,  *P* < 0.0001), and IL-8 (*r* = 0.799, 95% CI 0.579 to 1.075,  *P* < 0.0001).

Plasma levels of novel and traditional inflammatory mediators were measured in ESHF patients (pre-LVAD group) before and one month after LVAD implant (Figure 2). While sST2 did not show any modification, IL-33 reached lower levels after one month with respect to its preimplant values.

### 3.4. Relation with Outcome Indices

The clinical course of ESHF patients (pre-LVAD group) was evaluated considering the outcome indices.

Only cardiac expression of ST2, assessed at preimplant, showed a positive correlation with length of ICU, hospitalization and 1-week-tSOFA score (Figures 3(c), 3(d), and 3(e)). Moreover, cardiac expression of ST2 was higher in patients with composite critical outcome than in those without composite critical outcome (Figure 3(f)).

Accordingly, plasma levels of sST2 tended to be correlated with length of hospitalization (*r* = 0.453, 95% CI −0.192 to 1.808,  *P* = 0.104).

No inflammatory mediators showed any differences between patients who died after LVAD implant compared with those who survived over 1 month.

## 4. Discussion

Recent studies suggest that inflammation plays a role in the progression of HF [1–6]. Since clinical trials of anti-inflammatory therapies, such as anti-TNF-*α* approaches, have to date failed to show benefit in HF patients [6], a better definition of the role of inflammation in HF and new therapeutic targets are urgently required from basic research. The present study shows for the first time in a human model of HF that patients with ESHF presented differentially expressed levels of ST2/IL-33 as well as conventional inflammatory mediators, in both plasma and cardiac tissue, and that these modifications are corrected by mechanical unloading through LVAD support.

In this study, lower tissue expression of ST2 and IL-33 was found in cardiac tissue of patients undergoing LVAD support compared to more stable patients undergoing heart transplantation on medical therapy only, indicating that their decrease could be involved in the worsening of cardiac function in these groups of ESHF patients [1–6]. These data are in tune with previous reports indicating that IL-33/ST2 signaling has protective effects in regulating the myocardial response to pressure overload [9, 10, 20]. ST2 mRNA is highly induced in cardiac myocytes following mechanical strain or treatment with IL-1*β*, and serum sST2 levels transiently increased in mice subjected to coronary artery ligation [20]. IL-33 was also shown to be a biomechanically induced protein in the heart, synthesized by cardiac fibroblasts which antagonized angiotensin II and phenylephrine-induced cardiomyocyte hypertrophy [9]. Furthermore, IL-33 treatment reduced hypertrophy and fibrosis following transverse aortic constriction (TAC) in vivo via a reduction in NF*κ*B activation. In contrast, ST2^−/−^ mice had more left ventricular hypertrophy, more chamber dilation, reduced fractional shortening, more fibrosis, and impaired survival compared with wild-type littermates following TAC. IL-33 can also reduce cardiomyocyte apoptosis, decrease infarct and fibrosis, and improve ventricular function in vivo via suppression of caspase-3 activity and increased expression of the inhibitor of the apoptosis (IAP) family of proteins [10].

The involvement of ST2/IL-33 pathway in cardiac protection is confirmed by the increase in their levels by mechanical unloading after LVAD support, which was able to restore comparable levels to those observed for the heart transplant group of patients. We might speculate that the correction of ST2/IL-33 expression could be related to the reverse remodeling process induced by mechanical unloading. This is also supported by the positive association of ST2 with the classical molecular markers of inflammation as well as with tSOFA score, a clinical indicator of systemic inflammatory condition [17].

In order to study the possible contribution of myocardial proteins to their circulating levels, both IL-33 and sST2 were measured in peripheral circulation. We found that baseline cardiac ST2 positively correlated with its soluble isoform and did not show any modification after 1 month of LVAD support. These data might confirm the cardiac production of soluble sST2. In agreement with these results, ST2 production was previously demonstrated in vitro from a different type of cardiac cells [21, 22]. However, this remains an important issue which must be further clarified because discordant data are still present [23].

On the other hand, in experimental animal models, sST2 blocked antihypertrophic effects of IL-33, indicating that sST2 functions in the myocardium as a soluble decoy receptor [10]. This action together with the aforementioned cardiac production of sST2 could explain the negative prognostic value of this biomarker in individuals with HF. Several studies have since demonstrated the prognostic value of measuring serum sST2 in various cardiovascular diseases, showing that high baseline levels of sST2 were a significant predictor of cardiovascular mortality and HF [24, 25]. Accordingly, we showed that cardiac ST2 expression positively correlated with indices of outcome and sST2 tended to be positively correlated with length of hospitalization. These results could suggest that ST2 produced by cardiac muscles might also act in peripheral circulation as soluble decoy receptor for IL-33 thus reducing its protective effect [10]. Consequently, this action of ST2 could explain its negative prognostic role in HF, as confirmed by our results.

Conversely, before LVAD implant cardiac IL-33 was negatively related with its plasma concentration that resulted significantly decreased after 1 month compared to its values before LVAD support, suggesting a different regulatory mechanism for IL-33. IL-33 appears to be a cytokine with dual function, acting both as a traditional cytokine through activation of the ST2L receptor complex and as an intracellular nuclear factor with transcriptional regulatory properties. It is able to activate cells of both the innate and adaptive immune system, to function as an alarmin alerting the immune system to necrosis, and depending on the disease can either promote the resolution of inflammation or drive disease pathology [26]. So far, serum or plasma IL-33 has not been measured in cardiovascular disease. According to the results of this study, while IL-33 levels are elevated in atopy and in some rheumatological diseases, its levels in cardiovascular disease are likely to be low possibly due to elevated sST2 levels [27].

A further important result emerged from this study is that cardiac ST2 expression levels were significantly lower in patients with ischemic (IHD) than in patients with idiopathic dilated etiology (IDC). Previous data clearly suggest that the beneficial effects of LVAD were in part related to the etiology of the underlying HF. In fact, ESHF related to myocardial infarction has been shown to have much poorer response to LVAD therapy than nonischemic IDC [28]. The response of IDC to LVAD therapy is of particular interest because the myocardium is dysfunctional yet viable, unlike end-stage IHD. Accordingly, in this study ST2 resulted in an important molecule involved in regulation of HF, able to distinguish different HF etiologies. Moreover, the high levels of cardiac ST2 in IDC patients highlighted and confirmed its involvement in the mechanism of heart recovery.

Finally, results from this study provided further insights regarding the role of classical inflammatory mediators in HF. As with the IL-33/ST2 pathway, classical inflammatory mediators such as IL-6, IL-8, and TNF-*α* were low in less stable HF patients (pre-LVAD group) and increased after LVAD support to a level comparable to that of patients directly undergoing heart transplantation with only medical therapy (HT group). In spite of their well-documented role as proinflammatory cytokines, these molecules showed a negative role in HF progression and were positively involved in the reverse remodeling process by LVAD, eventually suggesting a compensatory effect to the adverse remodeling process of HF. In agreement with these results, recent studies hypothesized that temporally regulated activation and suppression of inflammation may be critical for achieving effective cardiac repair, indicating a paradoxical role of inflammation in cardiac repair [1]. Moreover, these findings highlighted the complexity of cytokine signaling and may provide explanations for the lack of clear results in clinical studies of cytokine-target therapies.

The relatively few studies conducted to date into the specific role of IL-33/ST2 in experimental human models limit definitive conclusions about the role that IL-33 and ST2 may play in patient outcome. However, the findings about classical inflammatory cytokines may suggest a pivotal role for these cytokines not only in regulating the inflammatory response but also in the critical balance among fibrogenesis, tissue regeneration, and cell death (apoptosis and/or necrosis). Thus, it could be possible to speculate that a fine tuning of both novel and classical inflammatory mediators, including a chemical, temporal, and spatial relationship and regulation, may occur and differentially contribute to the final outcome of LVAD patients, accounting as a major determinants of tissue remodeling.

The main limitation of this study is represented by the low number of patients. However, the internal control (HT group) and the post-LVAD group operated by collecting in the same patient myocardial tissue at HT time from both LV and RV allowed a better interpretation of the results in this limited sample size. Moreover, this low sample size made it difficult to assess the impact of different clinical variables (i.e. therapies, risk factors, etc.) on the modulation of IL33/ST2 pathway.

## 5. Conclusions

Our study is the first investigation in which the IL-33/ST2 pathway was studied in the in vivo setting represented by a human model of HF. Our results indicated that novel and traditional mediators of inflammation are involved in the decline of cardiac function in ESHF patients as well as in the process of reverse remodeling induced by LVAD support, supporting the value to cytokine measurements in HF patients. It remains to clarify specifically in human cardiac tissue which kind of cells were responsible for these modifications and how they were regulated at the molecular level. These data suggested that understanding inflammatory regulation in a more detailed manner is the key to achieving more effective cardiac repair and regeneration by new and more specific therapeutic strategies.

Manipulation of the IL-33/ST2 pathway represents a promising new therapeutic approach for treating or preventing various disorders in which inflammation is a critical process. To date, several approaches have been proven to modulate IL-33/ST2 signaling, addressed to its cardioprotective activity [29]. However, due to the involvement of the IL-33/ST2 system in a variety of processes, further studies are needed for its potential clinical use.

## Figures and Tables

**Table 1:** Analytical details of gene primers for real-time PCR analysis.

		Sequence	GenBank, accession number	Length (bp)	Temp (°C)	Efficiency (%)	*R* ^2^
ST2	Forward	CTCCAAGTTCATCCCCTCT	NM_000877.2	110	60	103.1	0.991
	Reverse	GATCCAAAACCCCATTCTGTT					
IL-33	Forward	GGAGTGCTTTGCCTTTGGTA	NM_033439	140	60	100.2	0.991
	Reverse	TCATTTGAGGGGTGTTGAGA					
IL-8	Forward	CCAAGCTGGCCGTGGCTTCTC	NM_000584	185	64.5	100.6	0.997
	Reverse	TGTGTTGGCGCAGTGTGGTCC					
IL-6	Forward	AGCGCCTTCGGTCCAGTTGC	NM_000600	121	64.5	100	0.999
	Reverse	GTGGCTGTCTGTGTGGGGCG					
TNF-*α*	Forward	TCCTCAGCCTCTTCTCCTTC	NM_000594.2	279	58	119.8	0.997
	Reverse	CCAGCTGGTTATCTCTCA					
RPL13a	Forward	CGCCCTACGACAAGAAAAAG	NM_012423	206	60	104.6	0.999
	Reverse	CCGTAGCCTCATGAGCTGTT					
PPIA	Forward	CTTGGGCCGCGTCTCCTTCG	NM_021130	285	60	103.4	0.998
	Reverse	TTGGGAACCGTTTGTGTTTGGGGC					
YWHAZ	Forward	ATGCAACCAACACATCCTATC	NM_00113572	178	60	95.3	0.997
	Reverse	GCATTATTAGCGTGCTGTCTT					

**Table 2:** Clinical features of ESHF patients according to sample groups.

	pre-LVAD group (*n* = 22)	HT group (*n* = 7)	*P* value*	Post-LVAD group (*n* = 6)	*P* value^#^
Age, years	58 (48–64)	55 (46–62)	0.459	44 (41–51)	0.031
Male gender, *n* (%)	19 (86)	5 (71)	0.569	6 (100)	1.000
Etiology, *n* (%)			0.202		0.673
IDC	12 (55)	6 (86)		4 (67)	
IHD	10 (46)	1 (14)		2 (33)	
Treatments, *n* (%)					
ACE-I and/or ARB	13 (59)	5 (71)	0.677	3 (50)	1.000
Beta-blockers	16 (80)	5 (71)	0.633	4 (67)	0.596
Statins	6 (27)	2 (29)	1.000	—	0.284
Antiplatelet agents	12 (54)	2 (29)	0.390	6 (100)^§^	0.062
Inotropic support	11 (50)	1 (14)	0.187	2 (33)	0.655
Creatinine, mg/dL	1.08 (0.90–1.53)	1.32 (1.00–1.78)	0.313	0.95 (0.83–1.48)	0.599
t-Bil, mg/dL	1.43 (0.55–1.90)	0.76 (0.48–1.14)	0.212	0.73 (0.31–1.34)	0.199
NT-proBNP, ng/L	2838 (1371–6042)	2389 (840–5762)	0.522	599 (158–1036)	0.007
LVEF, %	23 (19–25)	28 (20–29)	0.220	32 (20–33)	0.104
LVEDV, mL	202 (173–291)	228 (206–300)	0.185	239 (197–259)	0.820
LVEDD, mm	67 (57–71)	70 (68–79)	0.132	68 (60–75)	0.633
RAP, mmHg	5 (3–10)	3 (2–5)	0.074	5 (2–10)	0.969
PCWP, mmHg	25 (17–31)	11 (4–20)	0.019	10 (7–21)	0.023
CI, L/min/m^2^	1.7 (1.4–2.2)	2.0 (1.5–2.7)	0.362	2.3 (1.9–2.8)	0.085
PAPs, mmHg	55 (42–63)	28 (19–42)	0.012	29 (21–33)	0.006

Data are expressed as median (I–III interquartile range) or frequency (percentage).

ACE: angiotensin converting enzyme; ARB: angiotensin receptor blockers; CI: cardiac index; IDC: idiopathic dilated cardiomyopathy; IHD: ischemic heart disease; LVEDD: left ventricular end-diastolic diameter; LVEDV: left ventricular end-diastolic volume; LVEF: left ventricular ejection fraction; PAPs: systolic pulmonary arterial pressure; PCWP: pulmonary capillary wedge pressure; RAP: right atrial pressure; t-Bil: total bilirubin.

*P values pre-LVAD group versus HT group; ^#^ *P* values pre-LVAD group versus post-LVAD group; ^§^ *P* < 0.05 versus HT group.
